# How Large is the Potential of Brain Dead Donors and what Prevents Utilization? A Multicenter Retrospective Analysis at Seven University Hospitals in North Rhine-Westphalia

**DOI:** 10.3389/ti.2023.11186

**Published:** 2023-05-12

**Authors:** Jan Sönke Englbrecht, Daniel Schrader, Holger Kraus, Melanie Schäfer, Dirk Schedler, Friedhelm Bach, Martin Soehle

**Affiliations:** ^1^ University Hospital Münster, Münster, Germany; ^2^ University Hospital Düsseldorf, Düsseldorf, Germany; ^3^ University Hospital Essen, Essen, Germany; ^4^ University Hospital RWTH Aachen, Aachen, Germany; ^5^ University Hospital Cologne, Cologne, Germany; ^6^ Protestant Hospital Bethel (EvKB), Bielefeld, Germany; ^7^ Medical School OWL, Bielefeld University, Bielefeld, Germany; ^8^ University Hospital Bonn, Bonn, Germany

**Keywords:** organ donation, brain death, consent, opt-in, decision-maker

## Abstract

Organ donation after brain death is constantly lower in Germany compared to other countries. Instead, representative surveys show a positive attitude towards donation. Why this does not translate into more donations remains questionable. We retrospectively analyzed all potential brain dead donors treated in the university hospitals of Aachen, Bielefeld, Bonn, Essen, Düsseldorf, Cologne and Münster between June 2020 and July 2021. 300 potential brain dead donors were identified. Donation was utilized in 69 cases (23%). Refused consent (*n* = 190), and failed utilization despite consent (*n* = 41) were reasons for a donation not realized. Consent was significantly higher in potential donors with a known attitude towards donation (*n* = 94) compared to a decision by family members (*n* = 195) (49% vs. 33%, *p* = 0.012). The potential donor´s age, status of interviewer, and the timing of the interview with decision-makers had no influence on consent rates, and it was comparable between hospitals. Refused consent was the predominant reason for a donation not utilized. Consent rate was lower than in surveys, only a known attitude towards donation had a significant positive influence. This indicates that survey results do not translate well into everyday clinical practice and promoting a previously documented decision on organ donation is important.

## Graphical Abstract

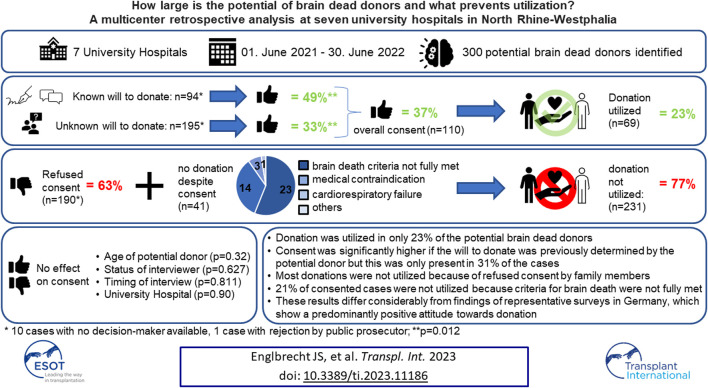

## Introduction

The number of utilized donations after brain death (DBD) has remained at a low level in Germany in recent years and is comparatively low in contrast to other countries [1, 2]. Spain realized 40.2 donations per one million inhabitants in 2021, the United States of America 41.9 and Germany 11.2, respectively [1]. There was a remarkable drop in utilized DBD in 2012 because of an organ allocation scandal in Germany [3] and donations since then have not returned to previous levels [2].

On the other hand, results from representative surveys by the Federal Centre for Health Education (“Bundeszentrale für gesundheitliche Aufklärung” - BZgA) show a constantly positive attitude towards organ donation, which was not significantly influenced by the scandal [3]. In 2020, 82% of the German population had a positive attitude towards donation. A share of 62% of the respondents said they had already made a decision and of these, 71% would agree to donate [4]. In its annual report 2021, the German Organ Procurement Organization (“Deutsche Stiftung Organtransplantation” - DSO) states that the rate of refusals among a total of 1,280 “qualified organ donors” (defined by the DSO as deceased persons in whom brain death has been determined and medical contraindications are absent) is only just under 19% [2].

The reasons for the discrepancy between the positive attitude and the low level of refused consent on the one hand, and the low rate of utilized donations on the other, remain unclear. This has been described in other countries with few organ donors, such as Switzerland [1, 5]. The country-specific legislation is one factor, that is repeatedly discussed [6–8]. Consent to donation in Germany is based on an opt-in system. In May 2012, an amendment to the Transplantation Act was introduced. The decision solution (“Entscheidungslösung”) as a modification of the opt-in consent was established in August 2012. The population is regularly informed about organ donation by their health insurers and they receive an organ donor card [8]. There are no formal requirements if or how the will to donate is registered. This can be done by filling out the donor card, documenting the decision in an advance directive or communicate the will with family members or witnesses. If the will is unknown, family members are asked to decide in accordance with the presumed will of the donor or their own values [9]. Another aspect discussed to explain the low numbers is the fact, that only DBD is allowed in Germany, whilst donation after circulatory death (DCD) is refused by the German Medical Association (“Bundesärztekammer” – BÄK) [10, 11].

An inadequate identification of potential DBD, not considering to diagnose brain death, or disregard of a possible wish to donate organs in the context of end-of-life decisions could also contribute to the low numbers [7, 11–13].

Published preliminary data from our workgroup revealed, that consent is significantly dependent on whether and how the potential DBD has documented his will to donate. Highest consent rate is found when a will to donate is previously confirmed in writing by the potential DBD whereas it is lowest for a decision by family members if the will of the donor is unknown [14]. The present analysis intents to provide further answers on the question of how many potential DBD exist in the participating hospitals, how many donations can be utilized, and what factors influence consent and utilization of organ donation after brain death.

## Patients and Methods

### Identification of Potential Organ Donors and Inclusion Criteria

The Madrid resolution, introduced in 2011, defines a critical pathway for assessing the potential of deceased donation and for the identification of possible deceased donors. It describes among other things a definition of a potential DBD donor (“a person whose clinical condition is suspected to fulfill brain death criteria”) [15]. The BÄK-guideline on donor identification, which is mandatory for all physicians in Germany, describes a comparable definition of a potential DBD donor (“a patient with primary and/or secondary brain damage, who is mechanically ventilated and treated in an intensive care unit (ICU), who is eligible as organ donor according to medical assessment, in whom brain death is imminent, suspected to have already occurred or in whom brain death has already been diagnosed”) [16].

All potential DBD who met this definition and who were treated in the ICU of the University Hospitals of Aachen, Bielefeld, Bonn, Düsseldorf, Essen, Cologne and Münster between 1st June 2020, and 30th June 2021, were retrospectively included into the analysis. Identification and medical assessment of whether a patient was a potential DBD was made by the attending physician and supported by the inhouse transplant coordinator (“Transplantationsbeauftragter” - TxB), who was a mandatory participant in every case due to obligations by the BÄK-guideline [16].

Data were collected retrospectively from the medical files and from the records of the TxB. The completeness of the study cohort was confirmed with a computer program (“Transplant Check”) provided by the DSO, which retrospectively identifies all in hospital deaths of patients with primary and/or secondary brain damage from the patient data according to § 21 Hospital Remuneration Act (“Krankenhausentgeltgesetz”) (a law that legally regulates the charges for full and partial inpatient hospital services) [11].

### Parameters and Variables

It was evaluated if and how the potential DBD had previously defined his will to donate. If the will was unknown, it was evaluated if family members existed, who were authorized to decide about a potential donation according to the presumed will or their own values. Consequently, the number of consented and utilized donations, reasons for a donation not utilized despite consent and predefined variables potentially influencing consent to donation were recorded (Table 1).

**TABLE 1:** Parameters and variables recorded in the study cohort.

Parameter	Variable
Decision on organ donation	⁃ Consent
	⁃ Refusal
Organ donation utilized after consent	⁃ Yes
	⁃ No
Age of potential DBD	⁃ Years
Gender	⁃ Male
	⁃ Female
Decision-maker	⁃ Potential DBD
	⁃ Family members
	⁃ No decision-maker available
	⁃ Public prosecutor
Timing of interview with family members to evaluate consent	⁃ Before diagnosis of brain death
	⁃ After diagnosis of brain death
Status of Interviewer	⁃ Consultant
	⁃ Fellow
	⁃ Resident
	⁃ TxB
University Hospital	⁃ Aachen
	⁃ Bielefeld
	⁃ Bonn
	⁃ Düsseldorf
	⁃ Essen
	⁃ Cologne
	⁃ Münster

DBD, donation after brain death; TxB, inhouse transplant coordinator.

### Ethics Committee and Registration

The Ethics Committee of the University of Muenster approved the study protocol (file number 2021-801-f-S). In addition, the study was registered in the German Register of Clinical Trials (DRKS) (DRKS-ID of the study: DRKS00027854).

### Statistical Methods

Statistical analysis was performed using SigmaPlot 14.0 (Systat Software, Inc., San Jose, California, USA). Parameters were expressed as mean ± standard deviation in case of normal distribution, otherwise as median [25%; 75% percentile]. By means of a chi-square (χ^2^)-test, the consent rate for organ donation was analyzed as a function of the individual variables (Table 1) and a statistical significance was assumed at *p* < 0.05.

## Results

### Potential Brain Dead Donors

**FIGURE 1:**
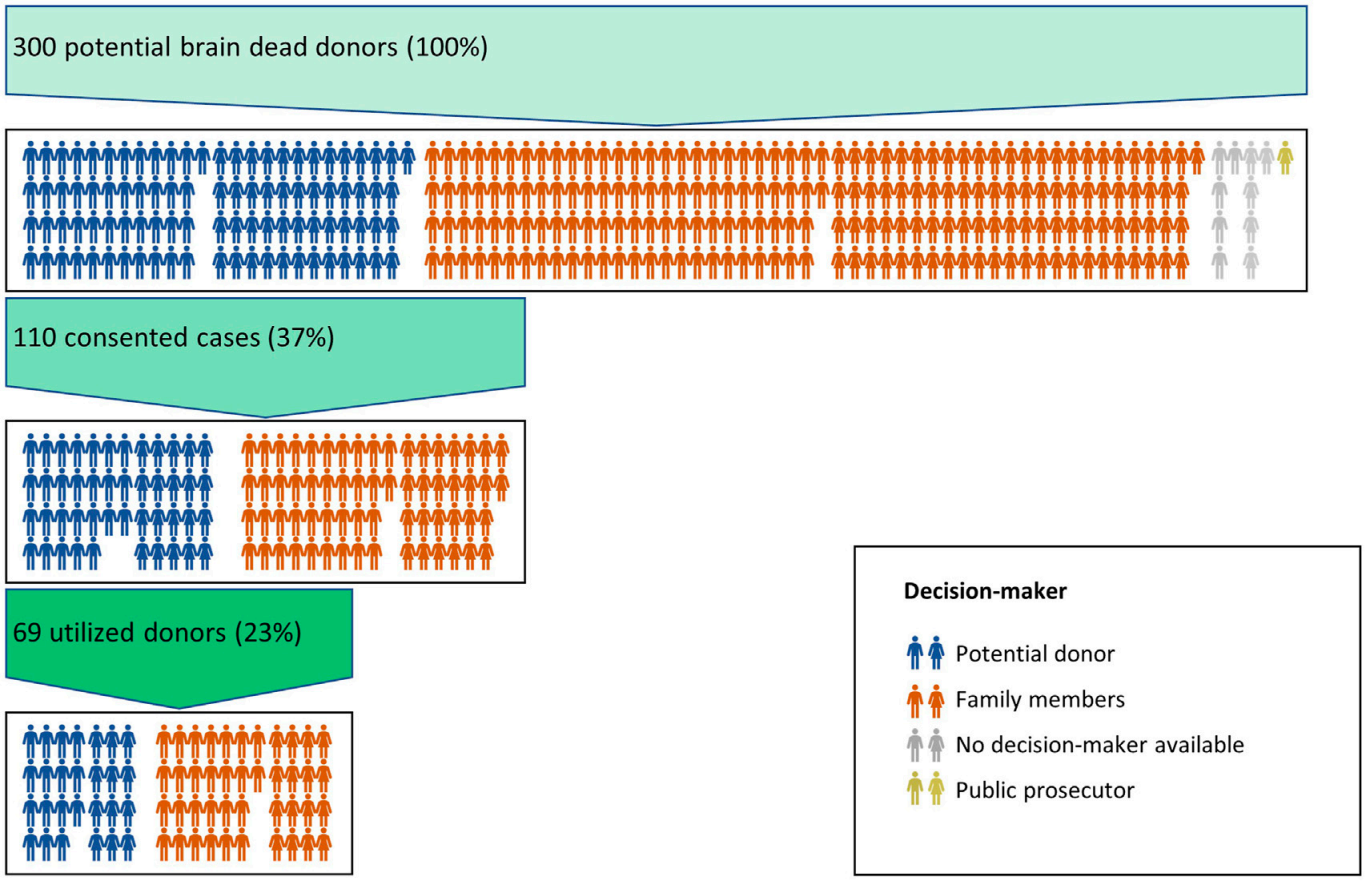
Overview of the study cohort. Each icon (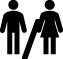) represents one male/female case. The colour of the icon indicates the basis of the decision about a donation in each case.

During the observation period, a total of 300 potential DBD (male: *n* = 152, female: *n* = 148) were identified in the seven university hospitals (Figure 1; Table 2).

**TABLE 2:** Demographics in the study cohort and basis of decision.

						Basis of decision^a^				
Cohort	N	Age^b^	Male	Female	*p*-value	Organ donor card	Advance directive/other document	Communicated orally	Family members	*p*-value
Potential DBD	300	61 [50; 77]	152	148		27	16	51	195	
Consented DBD	110 (37%)	60 [48; 71]	64 (42%)	46 (31%)	0.035	23	7	16	64	0.012
					0.063	((23+7+16) / (27+16+51)=49%)			33%	
Utilized DBD	69 (23%)	53.2 ± 19.6	41	28						

^a^ 10 cases with no decision-maker available, one case with refused consent by public prosecutor.

^b^ Age in years is displayed as average ± standard deviation in case of normal distribution, otherwise as median [25%; 75% percentile].

DBD, donation after brain death.

### Consent to Organ Donation

Overall consent to organ donation was found in 110 of the 300 cases (37%). The proportion of men was significantly higher in this collective (men/women: *n* = 64/46, *p* = 0.035), and the rate of consent tended to be higher in men than in women (42% vs. 31%, *p* = 0.063, Table 2). No consent could be obtained in the remaining 190 cases.

In 94 cases (31%), the potential DBD had previously defined his will to donate, leading to 46 consents (49%). In 195 cases (65%) the family members were to be involved because the will to donate was not determined by the potential DBD, resulting in 64 consents (33%). In ten cases, no decision-maker was available. In one case, consent was rejected by the public prosecutor. Consent rate was significantly higher, if the decision to donate was made by the potential DBD, compared to a decision by family members (49% vs. 33%, *p* = 0.012, Table 2).

### Utilized Organ Donation

Organ donation was utilized in 69 out of the 300 potential DBD (23%). In 41 cases, donation could not be utilized despite consent (14% of all cases or 37% of all consented cases). Reasons for this were preserved brain stem reflexes (*n* = 21) or inconclusive diagnosis of brain death (*n* = 2), medical contraindications (*n* = 14), or cardiovascular instability (*n* = 3). In one case, the reason was not documented.

### Variables Influencing Consent

The age of the potential DBD, the status of the interviewer, and the timepoint of the interview with family members about a decision when the will of the potential donor was unknown (before or after determination of brain death) had no influence on the consent rate, nor did it differ between the participating hospitals (Table 3).

**TABLE 3:** Consent rate as a function of parameters and variables.

Parameter	Variable	Potential DBD [n]	Consent [n]	%	*p*
Age [years]	0–9	7	4	57	0.32
	10–19	3	0	0	
	20–29	19	8	42	
	30–39	18	8	44	
	40–49	27	10	37	
	50–59	56	21	38	
	60–69	65	28	43	
	70–79	53	16	30	
	80–89	48	15	31	
	90–99	4	0	0	
Status of Interviewer	Consultant	151	61	40	0.627
	Fellow	31	9	29	
	Resident	44	15	34	
	TxB	64	25	39	
	No interview^a^	10	0	0	
Timing of Interview^b^	Before brain death	169	56	33	0.811
	After brain death	26	8	31	
University Hospital	Aachen	35	10	29	0.90
	Bielefeld	49	17	35	
	Bonn	47	16	34	
	Düsseldorf	58	22	38	
	Essen	43	17	40	
	Cologne	27	12	44	
	Münster	41	16	39	

^a^ No decision-maker available.

^b^ Interview with family members about the will to donate in 195 of the 300 cases, because the attitude towards donation was not previously determined by the potential donor.

DBD, donation after brain death; TxB, inhouse transplant coordinator.

## Discussion

The results of this retrospective analysis of utilized organ donations in potential DBD provide new explanations of the low number of donations in Germany, the apparent discrepancy to the positive attitude in representative surveys and the low refused consent rate published by the DSO. A donation could only be utilized in 23% of all identified potential DBD. In 37% of the consented cases, donation was nevertheless not possible. In 63% of all cases, consent was refused. Consent rate was significantly higher when the attitude towards donation was known, but only 31% of all potential DBD had previously determined their attitude towards organ donation. The age of the potential DBD, the status of the interviewer and the timing of the interview with family members to evaluate the will to donate had no significant influence on the consent rate, which was also comparable between the participating hospitals.

### Potential and Utilized DBD Donors

A total of 69 donations could be utilized in this cohort of 300 potential DBD (23%). Information about the total number of potential DBD in Germany is scare [11], partly due to the lack of epidemiologic studies and missing data about ICU-mortality [17]. Data from other countries show that the proportion of potential donors among deceased in the ICU ranges from 1.4% in Canada (with 36% utilized donors) [18], to 2% in Australia (33% utilized) [19] and 1.5%–2.4% in the Netherlands (26%–35% utilized) [20], respectively. Harvesting hospitals in Germany report annually to the DSO about their donation activities. The data analysis in these reports is based on numbers generated by the DSO tool “Transplant Check” (see Method section) [2, 11]. In 2020, a total of 14.164 death with documented brain damage were detected in all harvesting hospitals of NRW and 168 donations were utilized (1.2%) [21]. The corresponding numbers from the participating hospitals and the proportion of potential DBD donors identified in this study are shown in Table 4. Based on this data reference from 2020, the total proportion of potential DBD in this study was around 12% (ranging from 7% to 19% for each hospital) of all deceased with brain damage. This indicates that the low number of utilized donors is not a problem of failure to identify a potential DBD, at least in this cohort. However, it must be mentioned that the proportion could be different, if the number of potential donors were put in relation to all deceased in the ICU and not to all in hospital deceased with brain damage.

**TABLE 4:** Deceased with documented primary and/or secondary brain damage in 2020 in the participating hospitals, compared to the potential DBD donors identified in this study.

	Aachen	Bielefeld	Bonn	Düsseldorf	Essen	Cologne	Münster	Total
Deceased*	358	263	429	311	376	415	307	2,459
Contraindication to donation*	49	10	46	25	41	58	36	265
No mechanical ventilation*	94	79	136	81	97	131	89	707
No relevant brain damage*	40	37	28	18	31	81	9	244
Remaining cases with relevant brain damage*	175	137	219	187	207	145	173	1,243
Utilized donors*	5	10	3	6	10	15	5	54
Potential DBD in this study	35	49	47	58	43	27	41	300
Proportion of potential DBD/deceased	10%	19%	11%	19%	11%	7%	13%	12%

DBD, donation after brain death; *numbers from 2020 provided by the German Organ Procurement Organization [21].

In an older work, Wesslau et al found 600 utilized and 1,285 potential DBD in 2019 deceased with brain damage in the north east donor region of Germany between 2002 and 2005, indicating a higher proportion of utilized (47%) and potential DBD (64%) than in our cohort [17]. Notably, Wesslau et al defined potential DBD as “those for whom the diagnosis of brain death had been initiated and/or completed and no contraindications existed”. We used the definition of potential DBD according to the BÄK guideline on donor identification (see Method section). This could partially explain the lower rate of utilized DBD in our cohort because with our definition more potential DBD are identified. In our opinion this reflects a more precise definition of potential DBD and in turn a more realistic calculation of a representative consent rate, quite apart from the fact that the BÄK guideline also makes this definition mandatory [16]. The higher proportion of potential donors in Wesslau´s study might be due to fact, that they performed a prospective study, where only deceased in the ICU with a relevant brain damage were included by the attending physician rather than all deceased of a hospital with brain damage identified retrospectively by “Transplant Check.”

The most frequent reason for a donation not being realized in our cohort was refused consent in 190 cases (63%), including 48 (51%) refusals by the potential DBD and 131 (67%) refusals by family members, respectively. Wesslau et al found refused consent in 38% of potential DBD, but again this calculation was based on a different definition of potential DBD. Somewhat surprisingly, only refusals by family members were reported but no decisive information about refusals by the potential DBD are found in their analysis [17].

Additionally, an organ donation could not be utilized in 41 (37%) of the 110 consented cases in our cohort, in 17 of these cases due to the medical condition of the potential DBD. In 23 cases, the criteria for determining brain death were not fully met. Following the German legislation, an organ donation was thus not possible. In this constellation with severe brain damage, a therapy limitation due to an unfavorable prognosis usually leads to death from cardiovascular arrest within a short time. In many countries, organ donation would be permissible in such situations after planned therapy withdrawal (potential DCD donor). Twenty-one percent of all consented cases can be considered a relevant amount. At maximum utilization, the number of donors in this cohort would have increased from 69 (23%) to 92 (31%). Consequently, the fact that DCD is not possible may contribute to the overall low number of organ donations in Germany [6]. Although concerns have been raised that the success of implementing DCD would be at the expense of DBD donors [22], recent studies do not support these concerns [22–25]. A study from the United States identified almost 10.000 potential uncontrolled DCD donors per year, resulting in doubling the number of deceased donors, if maximally utilized [26]. In the United Kingdom, intended DCD donors (defined as patients who progressed to at least the organization of a theater team) increased from 1,187 between 2004 and 2009 to over 4,500 between 2009 and 2014 without a reduction in intended DBD donors [25].

### Consent to Organ Donation

Consent rate was 37% in this cohort, and a previously determined decision about donation was only found in 31% of all cases. These results differ considerably from surveys of the BZgA, in which 62% of the respondents stated that they had already made a decision and 71% of this group would agree to donate [4]. According to the 2021 DSO annual report, a consent to donate was achieved in 81% of the qualified DBD donors and in 42% the decision was made based upon a known will to donate [2].

The low number of realized donations despite the positive attitude to organ donation in surveys and the low rate of refused consent in reports from the DSO is repeatedly emphasized in the literature [7,11–13,27]. Subtly and sometimes explicitly, the reproach is voiced against hospitals that they are not sufficiently committed to increase the number of organ donations or that they do not identify eligible organ donors [11–13]. Based on the results of this study, this accusation seems – at least in part – not justified.

First, in this cohort, it was mainly family members who had to make the decision, and their consent rate was significantly lower. Lower rates of consent by family members when the donor’s will is unclear were also found previously by others [5, 28, 29]. It is obviously different to decide for oneself in a survey rather than for someone else, especially in the stressful situation where family members are asked to make a decision in an end-of-live setting, but only the presumed will or one’s own values can serve as the basis for this decision [30, 31]. Data from England and the USA have shown that knowledge about a person’s attitude to organ donation is one of the most important factors in consent by family members [28, 32]. In Switzerland, in 56% of the cases rejected by family members, it was stated they might have consented if there had been a documented will to donate [5]. Moreover, surveys on such a sensitive topic as the willingness to donate organs could lead to a bias in response behavior in favor of a perceived social desirability [33]. Whether the 2012 organ allocation scandal in Germany is still negatively influencing family members’ decisions because of a prevalence of mistrust in the transplant process cannot be answered from our data, but surveys suggest that public support for and confidence in organ donation and transplantation recovered quickly after the scandal [3].

Second, it is questionable to conclude from the consent rates reported by the DSO that similarly high consent rates must be found in everyday clinical practice. This is done in the literature to make a prediction about how many donations would be feasible [11]. In 3,132 organ donation-related contacts with the DSO in the year 2021, 1816 cases did not result in organ donation, in 29% of these cases because of a known refusal [2]. As contact with the DSO prior to conducting the diagnosis of brain death is optional, this refusal rate is not representative due to an unclear number of unreported cases [11]. For cases in which brain death has been diagnosed, there is a legal obligation to report to the DSO [34]. In these cases, the overall refusal rate according to the DSO was 19%, or 33% in the cases, where family members had to decide because of an unknown will of the potential DBD [2]. In our cohort, family members had to decide in 195 (65%) cases because of an unknown will and their rejection rate was 67% overall. However, in this cohort, the evaluation with family members about the will to donate was mostly carried out before brain death was confirmed (in 169 (87%) of the 195 cases). This approach is in accordance with the BÄK guideline on donor identification [16], recommending that a patient’s will for organ donation be explored at an early stage, at the latest when treatment limitations are being discussed. If a refused consent is communicated in this treatment phase, diagnose of brain death is often no longer performed, a palliative treatment concept is initiated, and the case is probably not reported to the DSO as organ donation is not possible. However, the DSO can only determine valid consent rates for cases with a completed diagnosis of brain death, as only then there is a legal obligation to report [34]. This could create a significant selection bias, as a negative attitude among potential donors who are not submitted to the diagnose of brain death may not be reported to the DSO [27]. Consequently, this can result in lower refused consent rates in their reports.

Third, in countries with opt-out consent, higher donor numbers can be achieved [35–37], although this positive effect is not demonstrable everywhere [38,39]. However, the opt-in consent used in Germany could have a negative impact on donor numbers, especially with regard to the significantly higher refusal rate by family members if the will of the potential donor is unknown [17]. Some politicians tried to address this issue with a legislative proposal that would introduce an opt-out system in Germany, but the majority of members of the German parliament voted against it in 2020 [40].

Finally, the DSO states the number of qualified donors in the 300 harvesting hospitals of North Rhine-Westphalia in 2020 to be 264 in total [41]. In this survey of seven university hospitals from North Rhine-Westphalia, 300 potential organ donors were identified, but not all of them were reported to the DSO, as a refused consent was already known before a pending determination of brain death. A lack of commitment in identifying potential organ donors cannot therefore be generally accused, although this is sometimes explicitly done [12, 13, 40].

### Age and Gender of the Donor

Mean age of the potential DBD was 61 and 60 years for the entire cohort and consented cases respectively. The DSO only provides numbers of age groups of utilized organ donations, with most donors aged between 16 and 55, but they give no information about the age of potential donors prior to the determination of brain death [2]. Others report an average age of potential donors of 55.1 years in Germany, and 50.5 years in consented organ donors, with a higher percentage of refusals in older age groups [17]. In this cohort, the age of the potential DBD had no significant influence on the consent rate. As expected, the cohort of utilized donations was younger than the group of potential DBD, presumably because medical contraindications are more common in older potential donors [17].

The proportion of male decedents with consent was significantly higher and the consent rate tended to be higher than for females. Others report significantly higher rates of consent among younger, male potential DBD [17], which is often also associated with a higher rate of consent after traumatic brain injury [32]. However, as the type of brain injury was not recorded in our retrospective survey, this hypothesis cannot be supported with the available data.

### Status of the Interviewer

The status of the interviewer when evaluating the will to donate with family members in the absence of a determined will had no significant influence on the consent rate in this cohort. Others could show that a combined approach by hospital staff and coordinators from an organ procurement organization resulted in the highest consent rate [42]. The United Kingdom provides specialist nurse training programs to train the communication with family members of potential organ donors [43]. Higher consent rates can be achieved when these specialists are involved in the decision-making process with family members [44].

Decision-making is usually a longer process with several communications with family members. In this retrospective study, only information on the conversation in which the decision was finally documented was collected. Since there is no information about any conversations that may have taken place before this process, the results of this cohort should not be over-interpreted. However, it seems generally undisputed that staff who are trained in dealing and communicating with family members of potential donors achieve higher consent rates. The status of the interviewer seems to be secondary in this respect [17, 42].

### Timing of Interview

In this cohort, the consent rate was comparable if the interview with the family members to evaluate possible consent in the absence of a known will of the potential DBD was conducted before or after the diagnosis of brain death. Other studies have also shown that the timing of the interview had no relevant influence on the consent rate [45]. There are only indications suggesting that there is a negative influence on consent if the question about organ donation is asked directly in the context of death notice or notification of the completed brain death diagnosis [42].

### Treating University Hospital

Consent rate did not differ significantly between the participating university hospitals. Calculations of conversion rates (realized organ donations/contact rates with organ procurement organization) or realization rates (realized organ donations/qualified organ donors) are often used to assess a “donation commitment” of an individual hospital [12]. These calculations show considerable differences between hospitals [12, 40].

However, the basis of this calculation gives rise to discussion. Contact with the DSO is not bindingly defined (according to the DSO’s procedural instructions, contact is required if the potential DBD is “eligible for organ donation according to medical assessment” [34]). This makes an objective comparison between hospitals based on conversion rates impossible. It is also questionable to use the number of “qualified organ donors” as a basis for comparison. By definition of the DSO, a “qualified organ donor” is one who has been diagnosed brain dead and who has no medical contraindications to donation [2]. This means that a hospital´s commitment to realize a donation is not captured for a case where a potential DBD is identified in advance of a possible brain death, but due to a known refused consent, brain death is not diagnosed. In our cohort, 113 refused consents were transmitted by family members prior to a pending diagnosis of brain death. Thus, using realization rates as an indicator for the “donation commitment” of a hospital should be treated with caution.

### Limitations

In this retrospective study, relevant factors possibly influencing results may not have been completely recorded, especially in such a difficult field as organ donation (e.g., no information on religious affiliation, type of brain damage or interviews prior to the final decision). It also cannot be completely ruled out that a possible consent to donate was not recorded due to lack of information about it. Only data from university hospitals in North Rhine-Westphalia were collected. It is possible that the results are not representative for the whole of Germany, as donor numbers may vary depending on the regions and the level of care provided by the hospital [11, 13]. The high proportion of identified potential DBD donors found in this study may not be generalizable to all harvesting hospitals in Germany, in part because there is evidence that the TxB are often not involved in the donation process, particularly in smaller hospitals [11] and they are not always given sufficient time off from their other duties to support donor identification [46].

## Conclusion

Following the recommended definition of a potential DBD, a donation could only be utilized in 23% of all potential DBD. The refusal rate was remarkably higher than results from representative surveys would suggest. Consent was significantly higher when the attitude towards donation was known but this was only available in 31% of all cases. Most refusals were communicated by family members before a pending diagnosis of brain death. A notable number of consented cases could not be transferred into utilized donations. These results suggest that attitudes to organ donation found in surveys and refusal rates provided by the DSO can only be transferred to everyday clinical practice to a limited extent. A clear definition of whether to involve the DSO and a requirement to involve the DSO early in the donation process when indicated, in combination with using internationally standardized definitions could provide more valid data on donor potential and consent rates in Germany. Better support for the work of the TxB might increase identification of potential DBD, and enabling DCD could be a promising option to increase donations. Considering the high rate of refused consent by family members in the absence of a known will, the implementation of an opt-out system should be discussed, as recently suggested by the German Federal Minister of Health [47]. As long as opt-in consent is used, promoting the documentation of a will to donate is essential to increase donations in Germany.

## Data Availability

Datasets are available on request from the corresponding author. Requests to access these datasets should be directed to jan.englbrecht@ukmuenster.de.
